# Reproductive history differs by molecular subtypes of breast cancer among women aged ≤ 50 years in Scotland diagnosed 2009–2016: a cross-sectional study

**DOI:** 10.1007/s10549-022-06721-1

**Published:** 2022-09-18

**Authors:** Anushri Chitkara, Ines Mesa-Eguiagaray, Sarah H. Wild, Peter S. Hall, David A. Cameron, Andrew H. Sims, Jonine D. Figueroa

**Affiliations:** 1 Usher Institute, College of Medicine and Veterinary Medicine, University of Edinburgh, Edinburgh, EH8 9AG UK; 2 Cancer Research UK Edinburgh Centre, Institute of Genetics and Cancer, University of Edinburgh, Edinburgh, EH4 2XU UK

**Keywords:** Breast cancer, Molecular subtypes, Reproductive factors, ER status, HER2, Parity, Age at first birth, Time since last birth

## Abstract

**Background:**

The aetiology of breast cancers diagnosed ≤ 50 years of age remains unclear. We aimed to compare reproductive risk factors between molecular subtypes of breast cancer, thereby suggesting possible aetiologic clues, using routinely collected cancer registry and maternity data in Scotland.

**Methods:**

We conducted a cross-sectional study of 4108 women aged ≤ 50 years with primary breast cancer diagnosed between 2009 and 2016 linked to maternity data. Molecular subtypes of breast cancer were defined using immunohistochemistry (IHC) tumour markers, oestrogen receptor (ER), progesterone receptor (PR), human epidermal growth factor receptor-2 (HER2), and tumour grade. Age-adjusted polytomous logistic regression models were used to estimate odds ratios (OR) and 95% confidence intervals (CI) for the association of number of births, age at first birth and time since last birth with IHC-defined breast cancer subtypes. Luminal A-like was the reference compared to luminal B-like (HER2−), luminal B-like (HER2+), HER2-overexpressed and triple-negative breast cancer (TNBC).

**Results:**

Mean (SD) for number of births, age at first birth and time since last birth was 1.4 (1.2) births, 27.2 (6.1) years and 11.0 (6.8) years, respectively. Luminal A-like was the most common subtype (40%), while HER2-overexpressed and TNBC represented 5% and 15% of cases, respectively. Larger numbers of births were recorded among women with HER2-overexpressed and TNBC compared with luminal A-like tumours (> 3 vs 0 births, OR 1.87, 95%CI 1.18–2.96; OR 1.44, 95%CI 1.07–1.94, respectively). Women with their most recent birth > 10 years compared to < 2 years were less likely to have TNBC tumours compared to luminal A-like (OR 0.63, 95%CI 0.41–0.97). We found limited evidence for differences by subtype with age at first birth.

**Conclusion:**

Number of births and time since last birth differed by molecular subtypes of breast cancer among women aged ≤ 50 years. Analyses using linked routine electronic medical records by molecularly defined tumour pathology data can be used to investigate the aetiology and prognosis of cancer.

## Introduction

Breast cancer is the most common malignancy worldwide [1]. In Scotland, it constitutes 28.8% of all cancers among women, with 1 in every 9 women carrying a risk of developing it in her lifetime (https://www.scotpho.org.uk/).

Breast cancer has been classified into ‘intrinsic’ or molecular subtypes based on mRNA expression profiling that have different treatment and survival outcomes [2]. The characteristics of these molecular subtypes are largely distinguished by expression of various combinations of tumour markers such as oestrogen receptor (ER), progesterone receptor (PR), human epidermal growth factor receptor-2 (HER2) and Ki67 tumour proliferation marker. Although gene profiling is considered the gold standard for classification of molecular subtypes, given the cost and lack of genetic profiling in clinical practice, a similar classification defined by immunohistochemistry (IHC) staining is a well-accepted surrogate [3, 4]. The St. Gallen Expert Panel recommends using ER, PR and HER2, along with tumour grade as a proxy for Ki67 index in defining the subtypes when the latter is unknown [4]. Based on IHC characterisation, the molecular subtypes are: luminal A-like, luminal B-like (HER2−), luminal B-like (HER2+), HER2-enriched and triple-negative breast cancer (TNBC). As the luminal-like cancers (ER/PR+) express hormone receptors, they can be effectively treated with molecularly targeted hormone therapy and generally have better prognosis. Due to limited therapeutic targets, i.e. ER/PR or HER2 in TNBC, the most aggressive subtype, chemotherapy along with surgery are the primary treatment options [5, 6].

Reproductive factors have been well documented as key breast cancer risk factors with direct associations observed with early age at menarche, nulliparity, late age at menopause and first birth, and limited breastfeeding [7, 8]. Data also suggest that there is a temporal relationship with time since last birth, where a short-term increase in breast cancer risk is observed 3–5 years after last birth [9, 10], before a long-term protective effect of parity is observed compared to nulliparity.

Within Scotland’s renowned, high-quality routine electronic health records, the Scottish Cancer Registry (SMR06) is an excellent resource to investigate risk factors for cancer incidence. In Scotland, ER status data collection began in 1997, and PR and HER2 data collection started in 2009, almost a decade earlier than other registries in the UK. We have recently reported the high quality of these data and shown distinct temporal trends by molecular subtypes and observed increasing incidence of ER+ subtypes among women of screening age (50–70 years), among whom about half of all cases are diagnosed [11].

In this study, we aimed to assess whether there are differences in reproductive risk factors among invasive breast cancer cases diagnosed in Scotland using a ‘case–case’ approach. A case–case analysis compares the risk factor associations of breast cancer by comparing cases of a certain molecular subtype to cases of another subtype, without also describing risk factor patterns in women without breast cancer [12].

## Methods

### Data sources and study population

All persons that are residents of Scotland are registered with a GP practice (defined as residing in UK for 3 months or longer). Records from within the UK can be added for UK residents. There are no accurate records of emigrations outside the UK (Scotland), however within the registry there is a variable that indicates whether a woman emigrated (to England or a different country). Numbers were really small (< 20 cases for the whole study period had an embarkment date recorded). The Information Services Division (ISD) of Public Health Scotland holds population-level National Health Service (NHS) data for Scotland which can be deterministically linked using the Community Health Index (CHI) number, a unique patient identifier. Probabilistic linkage providing < 4% false positive and < 2% false negative linkage (https://www.scotpho.org.uk/publications/overview-of-key-data-sources/scottish-national-data-schemes/isd-linked-database). Incident primary breast cancer cases were identified using data from the Scottish Cancer Registry (https://www.isdscotland.org/Health-Topics/Cancer/Scottish-Cancer-Registry/) which attains an average of 95.4% breast cancer case ascertainment and is over 99% complete [13] (https://www.isdscotland.org/Quality-Indicators). All tumours diagnosed in women 20+ years of age, with a primary invasive breast cancer (defined on the basis of the International Classification of Diseases, 10th revision code of C50) between 1997 and 2016 were ascertained (https://www.ndc.scot.nhs.uk/National-Datasets) (https://www.isdscotland.org/Quality-Indicators).

Approval for the analysis was obtained from the Public Benefit and Privacy Panel (PBPP) of NHS Scotland, and analyses were conducted in the Scottish National Safe Haven (PBPP Reference Number 1718-0057).

#### Maternity data

CHI number and probabilistic matching were used to link cancer registry data (SMR06) to Scottish Morbidity Records maternity inpatient and day case records (SMR02) which was available from 1981. To improve completeness of maternity data, the study excluded women who were ≥ 16 years (i.e. already in their reproductive years) in 1981, resulting in a cohort of women born in 1966 or thereafter. Data on number of births, age at first birth and time since last birth, including both live births and stillbirths, were calculated. The number of births was derived from the number of maternity records each woman held in SMR02. The maternal age from the first maternity record for a parous woman was considered as her age at first birth. Time since last birth was calculated as the time from the most recent birth preceding a cancer diagnosis.

#### Molecular subtypes definition

The Scottish Cancer Registry (SMR06) records the receptor status for breast cancers using immunohistochemistry (IHC) staining for ER, PR and HER2, and for borderline IHC HER2 results the status based on fluorescence in situ hybridization (https://www.isdscotland.org/Cancer-Registration-Definitions). While ER status for breast cancer became available in SMR06 in 1997, recording of information on PR and HER2 status commenced only in 2009 (https://www.isdscotland.org/Cancer-Registration-Definitions). As we aimed to evaluate the subtypes based on ER, PR and HER2 status, we focused on cases diagnosed from 2009 onwards. Due to non-availability of data on Ki67 labelling index, tumour grade was employed as a proxy for distinguishing the luminal subtypes [4]. The outcome variable, breast cancer subtype, was derived from four variables in SMR06: ER status, PR status, HER2 status and histological grade of the tumour. The five subtypes were defined as: ‘luminal A-like’ [ER/PR+ HER2− grade 1 or 2], ‘luminal B-like (HER2−)’ [ER/PR+ HER2− grade 3], ‘luminal B-like (HER2+)’ [ER/PR+ HER2+], ‘HER2-overexpressed’ [ER-PR-HER2+], and ‘triple-negative breast cancer’ or ‘TNBC’ [ER-PR-HER2−]. SMR02 and SMR06 datasets were linked by ISD using a pseudonymised CHI.

The cohort was limited to women with complete data on IHC-defined molecular marker status and tumour grade. Further restricting to women born in 1966 or later and with a breast cancer diagnosis between 2009 and 2016, resulted in a cohort of women diagnosed at 50 years of age or younger.

### Statistical analyses

A total of 431 (10% of cases) had missing subtype data and were excluded from analyses. To provide finer adjustment for age, we used 5 year age categories in regression models (20–35, 36–40, 41–45, 46–50). Age distribution at diagnosis of breast cancer, number of births, age at first birth and time since last birth were computed for each breast cancer subtype. Pearson’s chi-square tests were used to test for differences between subtypes in the distribution of reproductive risk factors of interest. We determined the correlation of age at diagnosis and each reproductive risk factor by computing Spearman’s correlation coefficients [14]. Polytomous logistic regression models adjusted for age at diagnosis of breast cancer were used to estimate odds ratios (OR) and 95% confidence intervals (CI) with the most common subtype, luminal A-like, as the reference group. We tested for interaction of age using likelihood ratio test (LRT) in polytomous logistic regression models with and without interaction term for each reproductive risk factor of interest. Tests were considered statistically significant at the 5% level. Stata MP V14 (College Station, TX) was used for all analyses.

## Results

**Fig. 1:**
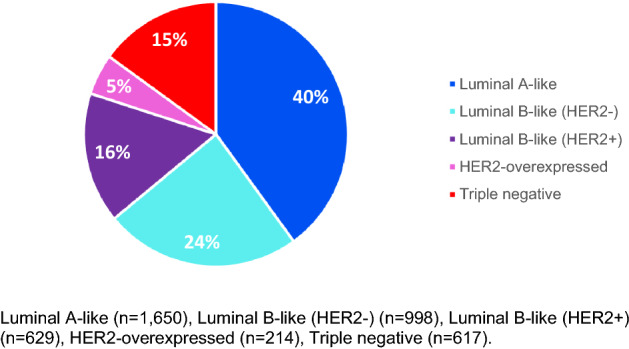
Distribution of breast cancer subtypes defined by immunohistochemistry and tumour grade among 4,108 women born after 1965 who had breast cancer diagnosed in Scotland between 2009 and 2016. Luminal A-like (*n* = 1650), luminal B-like (HER2−) (*n* = 998), luminal B-like (HER2+). (*n* = 629), HER2-overexpressed (*n* = 214), Triple-negative (*n* = 617)

The final study population included 4,108 women with breast cancer diagnosed at or below 50 years of age with data available to assign breast cancer subtype, after excluding 9.7% of the initial cohort with missing hormone status or tumour grade data (data not shown). There was a significant relationship between age at diagnosis and missingness of subtype, with patients 41–50 less likely to have missing subtype data (66.1% vs 59.6%), although the mean age was similar between those not missing subtype (mean age = 41.8 (SD = 5.3) and missing subtype (mean age = 40.9 (SD = 5.2). Luminal A-like was the most common type (40%) and HER2-overexpressed was the least common (5%, Fig. 1).

Distribution of age at diagnosis of breast cancer, number of births, age at first birth and time since last birth by the five breast cancer subtypes are presented in Table 1*.* Overall, 34% of breast cancers occurred in patients of age 40 years or younger and 66% between 41 and 50 years of age. The proportion of all luminal A-like tumours diagnosed in the age group 46–50 years was higher at 31.9% compared to the other subtypes being diagnosed in this age group (ranging from 19.7 to 22.6%). Women with luminal A-like subtype had the highest proportion of absence of birth records (assumed nulliparity of 30.5% or 69.5% with one or more birth records) and breast cancer diagnoses that were six or more years following their most recent birth (82.8%). Women with HER2-overexpressed and TNBC had higher proportions of one or more birth records (79.0% and 75.7%, respectively) and lower frequency diagnoses compared to luminal made six or more years after last birth (70.2% and 69.1%, respectively). Chi-square test revealed no statistically significant differences for age at first birth by subtype (Table 1). A significant correlation between age at diagnosis and time since last birth was observed (Spearman *R*^2^ = 0.66 *p* <0.001) as with age at first birth (Spearman *R*^2^ = 0.10) but not number of births (Spearman *R*^2^ = 0.19 *p* = 0.28).

**Table 1:** Descriptive characteristics of the cohort comprising of women born after 1965 and diagnosed with primary invasive breast cancer between 2009 and 2016 in Scotland stratified by surrogate molecular subtypes

Total number of cases (%):	Luminal A-like	Luminal B-like (HER2−)	Luminal B-like (HER2+)	HER2-overexpressed	TNBC	Overall
	1650 (40.2)	998 (24.3)	629 (15.3)	214 (5.2)	617 (15.0)	4108 (100.0)
Age at diagnosis (years)	N (%)	N (%)	N (%)	N (%)	N (%)	N (%)
20–35	122 (7.4)	133 (13.3)	105 (16.7)	37 (17.3)	119 (19.3)	516 (12.5)
36–40	283 (17.2)	239 (23.9)	152 (24.2)	55 (25.7)	149 (24.1)	878 (21.3)
41–45	719 (43.6)	400 (40.1)	248 (39.4)	76 (35.5)	223 (36.1)	1666 (40.6)
46–50	526 (31.9)	226 (22.6)	124 (19.7)	46 (21.5)	126 (20.4)	1048 (25.5)
*P value for heterogeneity between subtypes using chi-square test: < 0.001						
Number of births						
0	504 (30.5)	291 (29.2)	189 (30.0)	45 (21.0)	150 (24.3)	1179 (28.7)
1	370 (22.4)	215 (21.5)	151 (24.0)	53 (24.8)	153 (24.8)	942 (22.9)
2	524 (31.8)	337 (33.8)	202 (32.1)	77 (36.0)	215 (34.8)	1355 (33.0)
≥ 3	252 (15.3)	155 (15.5)	87 (13.8)	39 (18.2)	99 (16.0)	632 (15.4)
*P value for heterogeneity between subtypes using chi-square test: 0.111						

Number of parous cases (%):	1146 (39.1)	707 (24.1)	440 (15.0)	169 (5.8)	467 (15.9)	2929 (100.0)
	N (%)	N (%)	N (%)	N (%)	N (%)	N (%)
Age at first birth (years)						
< 20	144 (12.5)	89 (12.6)	44 (10.0)	18 (10.7)	73 (15.6)	368 (12.6)
20–24	246 (21.5)	162 (22.9)	88 (20.0)	43 (25.4)	117 (25.1)	656 (22.4)
25–29	348 (30.4)	195 (27.6)	149 (33.9)	49 (29.0)	117 (25.1)	858 (29.3)
30–34	268 (23.4)	169 (23.9)	100 (22.7)	45 (26.6)	101 (21.6)	683 (23.3)
≥ 35	140 (12.2)	92 (13.0)	59 (13.4)	14 (8.3)	59 (12.6)	364 (12.4)
*P value for heterogeneity between subtypes using chi-square test: 0.151						
Time since last birth (years)**						
≤ 2	76 (6.7)	71 (10.1)	53 (12.1)	20 (11.9)	70 (15.1)	290 (10.0)
3–5	120 (10.5)	116 (16.5)	70 (16.0)	30 (17.9)	73 (15.8)	409 (14.1)
6–10	279 (24.5)	186 (26.5)	115 (26.3)	41 (24.4)	121 (26.1)	742 (25.5)
> 10	663 (58.3)	329 (46.9)	199 (45.5)	77 (45.8)	199 (43.0)	1467 (50.4)

*P value for heterogeneity between subtypes using chi-square test: < 0.001

^*^Significant at 5% level

^**^Total counts (*N* = 2908) exclude parous women that did not have a pregnancy before diagnosis of breast cancer

Women with TNBC were significantly more likely to have at least one (relative to no birth records) in comparison to those with luminal A-like tumours (Table 2). Although based on fewer cases, a similar association was observed for women with HER2-overexpressed tumours who were more likely to have three or more births (relative to no birth records) when compared to women with luminal A-like tumours, in addition to a statistically significant test for trend across all subtypes. We observed a significant interaction with age at diagnosis and number of birth (LRT *p* = 0.05) hence we also present models adjusted for an interaction term. These results showed similar relationships, however estimates showed wider confidence intervals.

**Table 2:** Association of number of births among women born after 1965 and diagnosed with primary invasive breast cancer between 2009 and 2016 in Scotland by molecular subtypes adjusted for age at diagnosis with and without an interaction term for age at diagnosis

	Luminal B-like (HER2−) (*n* = 998)		Luminal B-like (HER2+) (*n* = 629)		HER2-overexpressed (*n* = 214)		TNBC (*n* = 617)	
	OR	95% CI	OR	95% CI	OR	95% CI	OR	95% CI
Number of births								
0	1.00	(reference)	1.00	(reference)	1.00	(reference)	1.00	(reference)
1	1.03	0.82–1.28	1.12	0.87–1.45	1.66	1.09–2.54	1.45	1.11–1.89
2	1.16	0.95–1.42	1.10	0.87–1.39	1.78	1.20–2.63	1.50	1.18–1.92
≥ 3	1.11	0.87–1.42	0.98	0.73–1.32	1.87	1.18–2.96	1.44	1.07–1.94
Age at diagnosis (years)								
20–35	1.00	(reference)	1.00	(reference)	1.00	(reference)	1.00	(reference)
36–40	0.76	0.56–1.03	0.62	0.45–0.86	0.60	0.37–0.96	0.52	0.37–0.71
41–45	0.50	0.38–0.66	0.40	0.30–0.54	0.32	0.21–0.50	0.30	0.23–0.41
46–50	0.39	0.29–0.52	0.27	0.20–0.38	0.27	0.17–0.44	0.23	0.17–0.32
Models with an interaction term between number of births and age at diagnosis								
Number of births								
0	1.00	(reference)	1.00	(reference)	1.00	(reference)	1.00	(reference)
1	1.46	0.83–2.56	1.23	0.69–2.22	2.00	0.83–4.82	1.65	0.92–2.95
2	2.72	1.57–4.71	1.87	1.05–3.34	2.48	1.02–5.98	2.46	1.38–4.40
≥ 3	1.74	0.95–3.91	0.95	0.48–1.85	2.59	0.99–6.74	1.91	1.00–3.64
Age at diagnosis (years)								
20–35	1.00	(reference)	1.00	(reference)	1.00	(reference)	1.00	(reference)
36–40	0.94	0.58–1.54	0.64	0.38–1.08	0.66	0.28–1.53	0.54	0.31–0.92
41–45	0.89	0.57–1.40	0.57	0.38–1.08	0.38	0.17–0.87	0.41	0.25–0.68
46–50	0.51	0.31–0.81	0.24	0.14–0.41	0.33	0.14–0.77	0.27	0.16–0.46

^*^Significant at 5% level

Referent subtype used here is luminal A-like breast cancer models included an interaction term between age at diagnosis and number of births

Table 3 shows case-case analysis for age at first birth by subtype. Luminal B-like HER2+ tumours compared to luminal A tumours were more likely to have a later age at first birth. In contrast, TNBC were less likely to have an older age at first birth compared to luminal A-like tumours. We did not observe statistical evidence of an interaction with age (LRT p = 0.40).

**Table 3:** Association of age at first birth among parous women born after 1965 and diagnosed with primary invasive breast cancer between 2009 and 2016 in Scotland by surrogate molecular subtypes adjusted for age at diagnosis

	Luminal B-like (HER2−) (*n* = 707)		Luminal B-like (HER2+) (*n* = 440)		HER2-overexpressed (*n* = 169)		TNBC (*n* = 467)	
	OR	95% CI	OR	95% CI	OR	95% CI	OR	95% CI
Age at first birth (years)								
< 20	1.00	(reference)	1.00	(reference)	1.00	(reference)	1.00	(reference)
20–24	1.08	0.77–1.51	1.18	0.77–1.79	1.41	0.78–2.55	0.94	0.66–1.36
25–29	0.89	0.64–1.22	1.36	0.92–2.02	1.10	0.62–1.95	0.66	0.45–0.91
30–34	1.00	0.72–1.39	1.21	0.80–1.82	1.32	0.74–2.38	0.72	0.51–1.07
≥ 35	1.17	0.81–1.71	1.53	0.97–2.43	0.91	0.43–1.91	0.96	0.63–1.47
Age at diagnosis (years)								
20–35	1.00	(reference)	1.00	(reference)	1.00	(reference)	1.00	(reference)
36–40	0.64	0.43–0.94	0.54	0.35–0.82	0.55	0.31–0.97	0.45	0.30–0.68
41–45	0.35	0.25–0.50	0.33	0.22–0.49	0.29	0.17–0.49	0.25	0.17–0.36
46–50	0.30	0.21–0.44	0.26	0.17–0.40	0.25	0.14–0.44	0.20	0.13–0.29

^*^Significant at 5% level

Referent subtype used here is luminal A-like breast cancer

When compared to the luminal A-like subtype, TNBC cases were significantly less likely to have last given birth > 10 years ago (relative to ≤ 2 years ago) (Table 4). Other subtypes did not show a clear association for time since last birth. We did not observe statistical evidence of an interaction with age (LRT p = 0.34).

**Table 4:** Association of time since most recent birth with among parous women born after 1965 and diagnosed with primary invasive breast cancer between 2009 and 2016 in Scotland who had their last birth prior to diagnosis by surrogate molecular subtypes adjusted for age at diagnosis

	Luminal B-like (HER2−) (*n* = 702)		Luminal B-like (HER2+) (*n* = 437)		HER2-overexpressed (*n* = 168)		TNBC (*n* = 463)	
	OR	95% CI	OR	95% CI	OR	95% CI	OR	95% CI
Time since last birth (years)								
≤ 2	1.00	(reference)	1.00	(reference)	1.00	(reference)	1.00	(reference)
3–5	1.21	0.79–1.84	0.98	0.61–1.56	1.14	0.60–2.17	0.79	0.51–1.24
6–10	1.06	0.71–1.57	0.87	0.56–1.35	0.89	0.47–1.66	0.74	0.49–1.13
> 10	0.95	0.63–1.41	0.76	0.48–1.18	0.87	0.46–1.64	0.63	0.41–0.97
Age at diagnosis (years)								
20–35	1.00	(reference)	1.00	(reference)	1.00	(reference)	1.00	(reference)
36–40	0.63	0.42–0.94	0.55	0.36–0.86	0.55	0.31–0.99	0.50	0.33–0.76
41–45	0.36	0.24–0.54	0.37	0.23–0.57	0.30	0.16–0.55	0.31	0.20–0.47
46–50	0.32	0.21–0.50	0.31	0.19–0.50	0.27	0.14–0.53	0.25	0.16–0.41

*Significant at 5% level

Referent subtype used here is luminal A-like breast cancer

## Discussion

Using Scottish cancer registry data linked to maternity health records, we show that parity, number of births and time since last birth to diagnosis of breast cancer differ by IHC-defined molecular subtypes of breast cancer among women ≤ 50 years of age at diagnosis of breast cancer. Breast cancer aetiology in younger women is not fully understood as few risk factors have been identified. Furthermore, few opportunities for early detection of breast cancer are available for younger women beyond genetic counselling for high-risk families.

Multiple reports and pooled analyses have recently evaluated IHC and mRNA expression profiling defined molecular subtypes of breast cancer and consistently show a positive association with parity for triple-negative or basal-like breast tumours [15–20]. Interestingly, significant differences in the incidence of breast cancer exist for different ethnic and racial groups that also frequently have different reproductive histories [21]. Consistent with these data, we also found evidence of heterogeneity in reproductive history across IHC-defined molecular subtypes of breast cancer in this Scottish cohort. Women with ER- tumours (HER2-overexpressed and TNBC) were more likely to have a higher number of births compared to women with luminal A-like subtype. Unlike ER- cancers, we did not observe heterogeneity in number of births between luminal B-like (HER2+) and luminal A-like, which concurs with other reports [22–25]. Time since last birth showed differential associations by subtype, where women with TNBC or luminal B-like (HER2+) were less likely than women with luminal A-like tumours to have a longer time between their most recent birth and diagnosis of breast cancer. Findings for TNBC correspond well with the existing studies [26, 27].

Parity confers a dual effect on the risk of breast cancer with an augmented risk observed in the initial years following pregnancy (3–5 years, or even up to 10–15 years) [28–30], possibly by stimulating the growth of cells that have undergone initial stages of malignant change and also due to the immunosuppressive effects of pregnancy [28, 31]. It is only subsequent to this phase that the protective effect of parity sets in [32, 33] owing to the differentiation of normal breast cells that have the potential to undergo malignant transformation. While this has been observed for ER+ breast cancers (luminal A-like) [8, 22, 34], an increased risk of ER- breast cancer continues to persist even in the longer term [25, 27, 35]. Our results did not observe significant differences across subtypes for age at first birth. However, TNBC cases were more likely to have a younger age at first birth when compared to luminal A-like cases (approximately 16% versus 12.5% patients for age at first birth < 20 years). A similar, statistically significant association has been reported by other studies [22, 23, 35–37]. Luminal B-like (HER2−) cases showed no statistically significant difference from luminal A-like for either of the three risk factors of interest even though studies have reported an inverse association with number of births and a positive association with age at first birth for this subtype [38, 39].

ER- breast cancers are less likely than ER+ breast cancers to be detected through screening [40], and predictive modelling of breast cancer risk has been proposed as possible solution for personalised medicine and risk stratified screening [41–43]. Modelling studies using UK data suggest such risk stratified screening approaches could reduce overdiagnosis, improve cost-effectiveness, while maintaining the benefits of screening [44].

The key strengths of our study are the high-quality longitudinal data collected within the Scottish Cancer Registry for the entire population, and the availability and high level of completeness of molecular marker and tumour grade data (≤ 10% missing data). Another strength of the study is the inclusion of women diagnosed at age 50 years or below. Although breast cancer is less common within this age range, the tumours are more aggressive with poor prognosis making it important to identify and implement effective approaches to prevention amongst this age group [45]. Moreover, breast cancer incidence appears to be increasing in younger age groups in recent years in Scotland [11] and other populations such as the United States [46].

Although this is one of the largest studies of breast cancer among young women, a limitation is the modest number of cases for rarer tumour subtypes, especially HER2-overexpressed (5% of all cases), potentially reducing the statistical power of analyses for these tumour subtypes. Our study did not assess incidence or risk of breast cancer, which would require comparisons to controls/general population. In addition, we cannot exclude some residual confounding by age at diagnosis since we did observe some association with age and missing subtype data. Future work including a comparison cohort of women not diagnosed with breast cancer would add further updated information about the role of reproductive history as a risk factor for breast cancer, including, in due course for whose breast cancer is diagnosed at older ages. Other limitations of our study were the potential for incomplete maternity records for women whose children were born outside Scotland, lack of availability of data for other factors such as breastfeeding as well as for a more detailed mRNA expression or mutation profiling of the cancers.

In conclusion, our data highlight the value of integrating molecular data from tumours with routinely collected health records data for understanding cancer epidemiology. There is scope for future analysis using the cancer registry linked to other datasets, including community prescription records, and primary care records, to provide more detailed information on the role and patterns of key risk factors and possible new aetiologic or prognostic factors for subtypes of breast and other cancers.

## Data Availability

Underlying data: All data used in the present study can be accessed by submitting an application to electronic Data Research and Innovation Service (eDRIS), a part of the Information Services Division of Public Health Scotland. More information on how to request access is available at https://www.isdscotland.org/eDRIS.

## References

[1] Sung H (2021). Global cancer statistics 2020: GLOBOCAN estimates of incidence and mortality worldwide for 36 cancers in 185 countries. Cancer J Clin.

[2] Perou CM (2000). Molecular portraits of human breast tumours. Nature.

[3] Cheang MCU (2009). Ki67 Index, HER2 status, and prognosis of patients with luminal B breast cancer. J Natl Cancer Inst.

[4] Goldhirsch A (2011). Strategies for subtypes—dealing with the diversity of breast cancer: highlights of the St Gallen International Expert Consensus on the Primary Therapy of Early Breast Cancer 2011. Ann Oncol.

[5] Tao L (2016). Occurrence and outcome of de novo metastatic breast cancer by subtype in a large, diverse population. Cancer Causes Control.

[6] O'Brien KM (2010). Intrinsic breast tumor subtypes, race, and long-term survival in the Carolina Breast Cancer Study. Clin Cancer Res.

[7] Newcomb PA (2011). Late age at first full term birth is strongly associated with lobular breast cancer. Cancer.

[8] Ma H (2010). Use of four biomarkers to evaluate the risk of breast cancer subtypes in the women's contraceptive and reproductive experiences study. Can Res.

[9] Nichols HB (2019). Breast cancer risk after recent childbirth: a pooled analysis of 15 prospective studies. Ann Intern Med.

[10] Tavani A (1999). Risk factors for breast cancer in women under 40 years. Eur J Cancer.

[11] Mesa-Eguiagaray I (2020). Distinct temporal trends in breast cancer incidence from 1997 to 2016 by molecular subtypes: a population-based study of Scottish cancer registry data. Br J Cancer.

[12] Martínez ME (2010). What can we learn about disease etiology from case-case analyses? Lessons from breast cancer. Cancer Epidemiol Biomark Prev.

[13] Brewster DH, Stockton DL (2008). Ascertainment of breast cancer by the Scottish Cancer Registry: an assessment based on comparison with five independent breast cancer trials databases. Breast.

[14] Stoltzfus J (2011). Logistic regression: a brief primer. Acad Emerg Med.

[15] Anderson KN, Schwab RB, Martinez ME (2014). Reproductive risk factors and breast cancer subtypes: a review of the literature. Breast Cancer Res Treat.

[16] Fortner RT (2019). Parity, breastfeeding, and breast cancer risk by hormone receptor status and molecular phenotype: results from the Nurses' Health Studies. Breast Cancer Res.

[17] Gaudet MM (2018). Pooled analysis of nine cohorts reveals breast cancer risk factors by tumor molecular subtype. Cancer Res.

[18] Ma H (2017). Reproductive factors and the risk of triple-negative breast cancer in white women and African-American women: a pooled analysis. Breast Cancer Res.

[19] Lambertini M (2016). Reproductive behaviors and risk of developing breast cancer according to tumor subtype: a systematic review and meta-analysis of epidemiological studies. Cancer Treat Rev.

[20] John EM (2018). Reproductive history, breast-feeding and risk of triple negative breast cancer: The Breast Cancer Etiology in Minorities (BEM) study. Int J Cancer.

[21] Figueroa JD (2020). Reproductive factors and risk of breast cancer by tumor subtypes among Ghanaian women: a population-based case-control study. Int J Cancer.

[22] Yang XR (2011). Associations of breast cancer risk factors with tumor subtypes: a pooled analysis from the Breast Cancer Association Consortium studies. J Natl Cancer Inst.

[23] Brouckaert O (2017). Reproductive profiles and risk of breast cancer subtypes: a multi-center case-only study. Breast Cancer Res.

[24] Devi CR, Tang TS, Corbex M (2012). Incidence and risk factors for breast cancer subtypes in three distinct South-East Asian ethnic groups: Chinese, Malay and natives of Sarawak, Malaysia. Int J Cancer.

[25] Kwan ML (2009). Epidemiology of breast cancer subtypes in two prospective cohort studies of breast cancer survivors. Breast Cancer Res.

[26] De Mulder H (2018). Breast cancer subtype and survival by parity and time since last birth. Breast Cancer Res Treat.

[27] Trivers KF (2009). The epidemiology of triple-negative breast cancer, including race. Cancer Causes Control.

[28] Lambe M (1994). Transient increase in the risk of breast cancer after giving birth. N Engl J Med.

[29] Liu Q (2002). Transient increase in breast cancer risk after giving birth: postpartum period with the highest risk (Sweden). Cancer Causes Control.

[30] Albrektsen G (2005). Breast cancer risk by age at birth, time since birth and time intervals between births: exploring interaction effects. Br J Cancer.

[31] Fornetti J (2012). Emerging targets for the prevention of pregnancy-associated breast cancer.

[32] Kelsey JL, Gammon MD, John EM (1993). Reproductive factors and breast cancer. Epidemiol Rev.

[33] Rosner B, Colditz GA, Willett WC (1994). Reproductive risk factors in a prospective study of breast cancer: the Nurses' Health Study. Am J Epidemiol.

[34] Setiawan VW (2009). Breast cancer risk factors defined by estrogen and progesterone receptor status: the multiethnic cohort study. Am J Epidemiol.

[35] Millikan RC (2008). Epidemiology of basal-like breast cancer. Breast Cancer Res Treat.

[36] Chen L (2016). Reproductive factors and risk of luminal, HER2-overexpressing, and triple-negative breast cancer among multiethnic women. Cancer Epidemiol Biomark Prev.

[37] Martinez ME (2013). Reproductive factors, heterogeneity, and breast tumor subtypes in women of Mexican descent. Cancer Epidemiol Biomark Prev.

[38] Ellingjord-Dale M (2017). Parity, hormones and breast cancer subtypes - results from a large nested case-control study in a national screening program. Breast Cancer Res.

[39] Horn J (2014). Reproductive history and the risk of molecular breast cancer subtypes in a prospective study of Norwegian women. Cancer Causes Control.

[40] Yen AM (2017). Initiators and promoters for the occurrence of screen-detected breast cancer and the progression to clinically-detected interval breast cancer. J Epidemiol.

[41] Howell A (2014). Risk determination and prevention of breast cancer. Breast Cancer Res.

[42] Lee A (2019). BOADICEA: a comprehensive breast cancer risk prediction model incorporating genetic and nongenetic risk factors. Genet Med.

[43] Pal Choudhury P (2020). Comparative validation of breast cancer risk prediction models and projections for future risk stratification. J Natl Cancer Inst.

[44] Pashayan N (2018). Cost-effectiveness and benefit-to-harm ratio of risk-stratified screening for breast cancer: a life-table model. JAMA Oncol.

[45] Brandt J (2015). Age at diagnosis in relation to survival following breast cancer: a cohort study. World J Surg Oncol.

[46] Lima SM (2020). Trends in parity and breast cancer incidence in US women younger than 40 years from 1935 to 2015. JAMA Netw Open.

